# Blockchain for Internet of Medical Things: A Technical Review

**DOI:** 10.1007/978-3-030-51517-1_22

**Published:** 2020-05-31

**Authors:** Fatma Ellouze, Ghofrane Fersi, Mohamed Jmaiel

**Affiliations:** 8 Digital Research Centre of Sfax, Sfax, Tunisia; 9 Institut Mines-Télécom, CNRS, Paris, France; 10 Université de Sherbrooke, Sherbrooke, QC Canada; 11 Digital Research Centre of Sfax, Sfax, Tunisia; 12 University of Sfax, Sfax, Tunisia; 13 ReDCAD laboratory, National School of Engineers of Sfax, University of Sfax, B.P. 1173, 3038 Sfax, Tunisia; 14 Higher Institute of Applied Sciences and Technology (ISSAT), University of Sousse, Sousse, Tunisia; 15 Digital Research Center of Sfax, B.P. 275, Sakiet Ezzit, 3021 Sfax, Tunisia

**Keywords:** Healthcare, Internet of Medical Things, Security, Blockchain

## Abstract

The Internet of Medical Things (IoMT) represents a network of implantable or wearable medical devices that continuously collect medical data about the patient’s health status. These data are heavy, sensitive and require high level of security. With the emergence of blockchain technology, researchers are focusing on using blockchain strategies to bring security to healthcare applications. However, such integration is very difficult and challenging due to the different requirements in these two technologies. We present in this paper a technical review of existing solutions applying blockchain technology on IoMT. We analyze these studies, discuss the proposed architectures and how they managed the integration challenges. The open issues regarding the application of blockchain over IoMT are also specified.

## Introduction

Recently, with the rapid development of wearable/implantable sensors and wireless communication, researchers are increasingly interested in improving the health sector in response to human needs by digitizing and decentralizing healthcare institutions and providing continuous and remote medical monitoring. Generated medical data are very critical and must be dealt with care to prevent any kind of data tampering. In this context, blockchain has emerged as the most secured, decentralized platform. It provides many powerful features without third party dealing including tamper-proof, immutability, traceability, data integrity, confidentiality and privacy.

Several research studies have identified blockchain effectiveness for the healthcare ecosystem. The papers [1, 8, 10, 11, 16] reviewed existing works related to using blockchain technology in healthcare to bring security. However, none of these works has focused on the integration of blockchain technology to the Internet of Medical Things. In this context, we propose our paper that reviews existing works related to the integration of blockchain with the IoMT and discuss the technical details of each work.

The remainder of this paper is structured as follows. Section 2 presents a detailed technical analysis of existing articles dealing with the integration of blockchain with IoMT. Section 3 provides an in-depth discussion based on our study and presents research gaps, while Sect. 4 concludes the paper.

## Internet of Medical Things (IoMT)-Blockchain Challenges

Certainly, blockchain technology is beneficial to the internet of medical things in terms of security. However, integrating both technologies is not trivial at all and is facing several challenges due to the conflicting requirements in these two technologies:

- **Processing:** Mining process and complex cryptography in blockchain are resource-hungry, demanding intensive computation and high energy consumption which cannot be afforded by resource-constrained IoMT devices that already suffer from resource shortage and energy limitations.
- **Storage:** IoMT devices generate huge amount of data with large flow. These data must be treated and stored in the blockchain to ensure their integrity which poses a significant challenge. In fact, blockchain technology relies on its nodes to provide a distributed storage which is not affordable by IoMT devices that have limited storage capabilities.
- **Mobility:** Blockchain was designed for a fixed network topology. However, implantable/wearable medical devices are in movement all the time which continuously change the topology.
- **Real Time:** IoMT applications are generally critical and require a real time and immediate response. Whereas, blocks creation is time consuming. In Bitcoin [15], 1MB per block is created every 10 min. Grouping these streams of data on blocks while respecting real time requirement is challenging.
- **Traffic Overhead:** Blockchain nodes communicate continuously to synchronize which creates significant overhead traffic. This is not affordable by bandwidth-limited IoT devices.

## Blockchain-Based Approaches in IoMT

We present in this section, the most recent researches that have applied blockchain on IoMT. We classify these researches according to the most leading technique used to integrate blockchain into IoMT.

### Ethereum-Based Contributions

In [12], a private Ethereum-based architecture is proposed to implement smart contracts in order to manage the users/devices requests and control access based on a set of attributes including the credentials, role and the domain. It uses IPFS for data storage. An interPlanetary File (IPFS) is used to store patient health records and devices technical information. The consensus mechanism is performed by a smart contract. The authors proposed a proof of medical stack (PoMS) as an alternative to PoS consensus model to protect smart contracts from malicious actions. PoMS allows stakeholders with huge amount of medical data presented as tokens to validate and create blocks.

A private blockchain-based system for medical data management has been proposed in [9]. It works on Ethereum smart contracts to manage data access permission between entities including patients, hospitals, doctors, research organizations and other stakeholders. The smart contract contains smart representations of medical records including permissions, record ownership metadata and data integrity. The medical record data are stored in external server (off-chain) and a cryptographic hash of the record is kept on the blockchain ensuring data integrity. The proposed system eliminates mining for simplification.

In [3], authors developed a cloud-based framework to monitor the progression of a neurological disorder disease using IoMT devices. They used cloud computing to store and process IoMT data and deploy Ethereum-based Blockchain network to securely exchange and share data between healthcare users. Smart contracts are employed to control users access to data in the cloud. No technical details about integrating blockchain in the system are presented.

In [6], authors proposed a permissioned blockchain-based architecture for secure remote patient monitoring. They used Ethereum to implement smart contracts in order to analyze data and send alerts to patient and healthcare providers. They proposed the use of Practical Byzantine Fault Tolerance (PBFT) as an alternative to PoW consensus model. The proposed architecture lacks techniques to meet challenges related to IoMT-Blockchain integration. And in SMEAD [13], an Ethereum-based architecture for remotely monitoring diabetes patients, smart contracts are used to manage access to data.

### Modified Consensus Protocol

In order to fit the IoMT specificities, some works like [12] have proposed to modify the consensus protocol. In [20], authors proposed a consortium blockchain-based architecture in order to record data generated from IoMT in a secure way while ensuring the patient’s privacy. The proposed architecture implements a patient agent software (PA) that defines the Blockchain functionalities. It is deployed on the Edge computing network to perform lightweight tasks and on a cloud server to provide tamper proof storage of the large volume of health data. The authors also proposed a modified PoS consensus which consists in choosing a leader for a group of nodes to validate and create the blocks. Smart contracts are used to manage health data including filtering clinically useless health data, generating alarm for some events, migrate data to the cloud if necessary, classify data and others. Compared to PoS, authors affirm that the modified PoS is more efficient in term of energy consumption and block generation time.

### Modified Cryptographic Technique

The authors in [14] use some features of the standard version of blockchain to provide privacy and data integrity when sharing IoMT data. They use the hashing technique and propose a newly encryption algorithm to encrypt the transactions containing personal and sensitive data about patients. The main advantage of this algorithm is its ability to cover large number of uniquely identified medical objects and its very low time complexity which fits the real time requirement of IoMT. All transactions are stored in a blockchain maintained by the healthcare providers.

In [5], Authors proposed a customized blockchain-based framework suitable for IoMT devices. First, the proposed blockchain is private: nodes must be certificated to be able to join the network and send transactions. Second, authors eliminate the POW consensus protocol. To deal with the high volume generated by IoMT devices, they group encrypted data in blocks and store the interconnected blocks in the cloud. The hashes of blocks are kept on the blockchain to ensure tamper proof storage. For anonymity and the authenticity of the user, they use a ‘A lightweight privacy-preserving ring signature scheme’ which allows a group of nodes to participate in the data signature. To secure data and ensure its integrity during the transmission and storage, the authors used double encryption scheme besides the digital signature. The data are encrypted using lightweight ARX algorithms and the key is encrypted using the receiver’s public key. To secure the transfer of public keys, authors proposed the Diffie-Hellmman key exchange technique. To meet scalability and network delay challenges, nodes are grouped in clusters. A cluster head is chosen to verify and store hash blocks, verify digital signatures and manage interactions between nodes in the cluster. The proposed work is not implemented and not evaluated.

In addition to their modified consensus protocol, authors in [20] proposed the ring signature as an alternative to the standard public key based digital signature to ensure patient privacy.

### Hyperledger-Based Contributions

In [2], the authors proposed an IoT-blockchain based architecture to allow healthcare remote monitoring. The architecture contains two types of blockchain: (1) Medical Devices Blockchain to store medical data generated by medical devices during treatment period, (2) Consultation Blockchain maintained by hospitals to permanently store patients records. The transactions are verified and validated using smart contracts (Chaincodes in Fabric) executed by endorsing peers following Practical Byzantine Fault Tolerance algorithm. The authors developed a user interface to visualize the patient health data.

### General Blockchain Concept Without Technical Specifications

In [7], the authors took benefit of tamper proof feature of blockchain to securely store and share IoMT data through patients and healthcare providers. The patient data are stored as strings in blocks in the blockchain and the IoMT data are stored in blocks in off-chain database like IPFS. Smart contracts are used to ensure the privacy and security of blockchain.

MedChain [18] is a consortium blockchain-based framework proposed to meet challenges related to efficiently sharing data streams continuously generated from medical sensors. This includes handling time-series data streams, managing mutable and immutable medical data, and allowing an efficient storage and sharing of big and sensitive data. The MedChain network includes two separate decentralized sub-networks: (1) Blockchain network to store immutable data including users identity, data digest, session and operation, and (2) P2P network to store mutable data that facilitates data query including the description of data and session. MedChain uses the BFT-SMaRt as a consensus protocol

BIoMT [17] is an optimized, lightweight blockchain-based framework proposed to meet security and privacy challenges in developing solutions for IoMT systems. The proposed architecture is made up of four stratum: (1) Device layer consists of IoMT devices and implements the Elliptic Curve Cryptography (ECC) [9] key establishment protocol and the identity-based credential (IBC) mechanism to provide decentralized privacy, (2) Facility layer for managing IoMT devices and providing unique identity based on their attributes, (3) Cloud layer that runs anonymization algorithms to allow an identity-free data analysis and storage, and (4) Cluster layer groups several entities including medical facilities, service providers, and cloud servers into clusters. Each cluster has a cluster head that manages communication with other cluster heads to decrease the network overhead and delay. This work does not provide any technical details. It is not implemented and not evaluated. In [4] and [19], a blockchain-based architecture is proposed to allow secure transmission and storage of large amount of sensitive data generated by IoMT.

## Discussion and Open Issues

Table 1 presents a classification of the existing contributions having integrated blockchain to IoMT (NM means Not Mentioned). Most of the proposed solutions are private blockchain-based and used Ethereum infrastructure thanks to its flexibility that is offered by the implementation of smart contracts for management purposes. Many issues have been treated when integrating blockchain with IoMT. For storing the big IoMT data, most of works [3] proposed an off-chain storage: Some researches [7, 12] proposed to use IPFS because of its distributed data structure. Other works [3, 5, 9, 17, 20] used the cloud computing to store encrypted data while keeping hash references of that data in the blockchain. Such solutions do not guarantee immutability which is the essential feature of blockchain. In fact, if data have been modified/altered, this will be detected thanks to their hash stored in the blockchain but not recovered as it is only stored in the cloud (centralized storage). Other studies proposed an on-chain storage without precising technical details about dealing with the huge amount of data streams generated by IoMT devices. In the other hand, healthcare applications require real time responses which require a fast consensus protocol. However, IoMT are constrained devices and produce huge amount of data. The majority of studied works [9] have eliminated the consensus protocol to meet IoMT requirements. Some authors [12] use smart contract to self-verify and self-execute transactions. These smart contracts are protected using a lightweight consensus mechanism. Some others [6] proposed a lightweight consensus protocol: Researchers in [12, 20] modified the PoS protocol to adapt it to the IoMT requirements, other works [5, 20] grouped the nodes in clusters and chose a header for each cluster to manage transactions between nodes, validate and create the blocks. For security requirements in healthcare domain, some existing works proposed solutions to manage and control access rights. The majority [3, 9, 12, 13] implement smart contracts to allow access to only authorized users based on some attributes of the IoMT ecosystem and their interaction with the users/stakeholders. Some other works [5, 14, 20] focused on maintaining patient privacy by proposing a lightweight privacy-preserving algorithms like ring signature scheme.

**Table 1.** Classification of researches applying blockchain in IoMT

Contribution	Framework	Type	Consensus	Storage	Digital signature	Smart contract	Use case
[20]	NM	Consortium	Cluster head verifies and adds blocks	Off-chain (cloud)	Ring signature	Analyze and manage data	Manage IoMT data
[14]	NM	Private	NM	On-chain (hospitals)	NM	NM	Privacy and data integrity preservation
[9]	Ethereum	Private	NM	Off-chain (external server)	NM	Smart representations of medical records	Manage IoMT data
[7]	Ethereum	Public	NM	Off-chain (IPFS)	NM	Manage interactions between patients and their data and doctors	Manage IoMT data
[12]	Ethereum	Private	Proof of medical stake	Off-chain (IPFS)	NM	Manage access control	Manage access control to IoMT data and devices
[2]	Hyperledger Fabric	Private	NM	On-chain (hospitals and medical devices)	NM	Verifying and validating transactions	Healthcare remote monitoring
[18]	NM	Consortium	BFT-SMaRt	On-chain	NM	NM	Manage IoMT data
[5]	NM	Private	Cluster head verifies and adds blocks	Off-chain (cloud)	Lightweight ring signature	Analyze IoMt data and control patient health	Remote patient monitoring
[17]	NM	Private	NM	Off-chain(cloud)	NM	NM	Manage IoMT data and devices
[3]	Ethereum	Private	NM	Off-chain(cloud)	NM	Manage access control	Monitor the progression of a neurological disorder
[4]	NM	Private	PoW	NM	NM	NM	Manage IoMT data
[6]	Ethereum	Private	PBFT	On-chain	NM	Analyze data and send alerts to patients and healthcare providers	Remote patient monitoring
[19]	NM	NM	NM	Hybrid	NM	NM	Manage IoMT data
[13]	Ethereum	NM	NM	NM	NM	Manage access control	Remote monitoring of diabetes patients

The literature review shows that there are some significant research gaps. There are several challenges that must be addressed to reach maturity and be efficient. These challenges include:

- **Lack of standards:** The proposed solutions are proprietary. They do not define standard protocols to adapt heterogeneous technologies and promote interoperability which prevent the adoption of such solutions. It is crucial to provide universal and platform-agnostic solutions that govern the interaction between IoMT devices, blockchain, cloud computing and end-users.
- **Programming Abstractions:** The integration of blockchain technology into the IoMT opens the way to many relevant applications in the health field. However, the adoption of such technology (Blockchain-IoMT) is complex and requires in-depth interdisciplinary knowledge from low-level including the management of IoMT devices and configuring blockchain to meet IoMT requirements, to high-level knowledge including sharing, storing and treating IoMT data. In this context, it is crucial to conceive an abstraction layer hiding all these complexities and to provide developers with new application programming interfaces (APIs) and middleware allowing them to easily implement decentralized and secure applications for healthcare using IoMT.
- **Limited Application Scope:** The majority of existing works are only focusing on healthcare applications related to remote patient monitoring and IoMT data management including data sharing and storage. It is crucial to conceive tracking applications that prevent counterfeit drugs and medical errors. In this context, the use of blockchain technology accompanied by the IoMT can be an effective solution to control the activity of doctors as well as for the management of the drug supply chain.
- **Lack of Technical Details:** The integration of blockchain with the IoMT is challenging. Most of existing solutions did not reveal any technical details. There is a need that researchers demystify all the technical details of the blockchain integration into IoMT.

## Conclusion

With the strict and severe requirements of security in the healthcare domain, several researches focused on adopting Blockchain in the Internet of Medical Things (IoMT). Majority of them were focusing on providing privacy, data integrity, confidentiality and authentication. They proposed different use cases including remote monitoring of patients (RMP) and medical data management. Our research review shows that the proposed solutions lack many technical details when integrating Blockchain in the IoMT. Majority of them did not deal with high volume of data streams generated by resource-constrained IoMT devices and did not propose technical modifications on the Blockchain architecture in order to feet these challenges.
